# Use of proton pump inhibitors and histamine-2 receptor antagonists and risk of gastric cancer in two population-based studies

**DOI:** 10.1038/s41416-020-0860-4

**Published:** 2020-05-05

**Authors:** Peipei Liu, Úna C. McMenamin, Brian T. Johnston, Peter Murchie, Lisa Iversen, Amanda J. Lee, Pauline A. J. Vissers, Chris R. Cardwell

**Affiliations:** 1 Centre for Public Health, Queen’s University Belfast, Belfast, UK; 2 Belfast Health and Social Care Trust, Belfast, UK; 3 Academic Primary Care, Institute of Applied Health Sciences, University of Aberdeen, Aberdeen, UK; 4 Medical Statistics Team, Institute of Applied Health Sciences, University of Aberdeen, Aberdeen, UK; 5 Department of Research, Netherlands Comprehensive Cancer Organisation (IKNL), Utrecht, The Netherlands

**Keywords:** Cancer epidemiology, Health sciences

## Abstract

**Background:**

Studies have shown increased gastric cancer risk in users of proton pump inhibitors (PPI) and histamine-2 receptor antagonists, questioning the safety of gastric acid suppression. Therefore, we conducted a case–control study within the Scottish Primary Care Clinical Informatics Unit (PCCIU) database and a cohort study in the UK Biobank.

**Methods:**

In PCCIU, five controls were matched to cases diagnosed in 1999–2011, and medications were determined from GP records. Odds ratios (OR) and 95% confidence intervals (CI) were calculated using conditional logistic regression. In the UK Biobank, medications were self-reported at cohort entry 2006–2010, and gastric cancer ascertained from cancer registries until 2014. Hazard ratios (HR) were calculated using Cox regression.

**Results:**

PCCIU contained 1119 cases and 5394 controls. UK Biobank contained 250 cases in 471,779 participants. PPI users had a higher gastric cancer risk in PCCIU and UK Biobank when applying a 1-year lag (adjusted OR = 1.49, 95% CI 1.24, 1.80; adjusted HR = 1.28, 95% CI 0.86, 1.90, respectively), but these associations were attenuated when using a 2-year lag (adjusted OR = 1.13, 95% CI 0.91, 1.40; adjusted HR = 1.15, 95% CI 0.73, 1.82, respectively).

**Conclusions:**

Overall, we observed little consistent evidence of an increased risk of gastric cancer with PPI use.

## Background

Gastric cancer is the fifth most common cancer worldwide with over a million newly diagnosed cases in 2018, and is the third leading cause of cancer mortality accounting for over 782,000 deaths globally.^1^ These high-incidence and mortality rates highlight the importance of preventing gastric cancer.

Proton pump inhibitors (PPI) and histamine-2 receptor antagonists (H2RA) are widely prescribed for the treatment of gastric diseases, such as peptic ulcer, dyspepsia, gastro-oesophageal reflux disease (GORD) and *Helicobacter pylori* (*H. pylori*) infection. The safety of long-term acid suppression has long been debated,^2^ and various mechanisms are of particular concern to gastric cancer risk. Acid suppression has been shown to cause excess blood gastrin levels,^3^ which have been suggested to lead to hyperplasia of enterochromaffin cells and ultimately gastric carcinoid formation.^4^ PPI and H2RA reduce the acid content of gastric acid causing hypochlorhydria, which can lead to an overgrowth of bacteria in the gut, reducing the absorption of nutrients and lowering protection against infections.^5,6^ Finally, it has been suggested that PPI could interact with *H. pylori*, leading to greater acid suppression causing *H. pylori* and *non-H. pylori* bacterial overgrowth that cause or exacerbate gastritis, something which is associated with increased gastric cancer risk.^7,8^

Consequently, the association between PPI and gastric cancer risk has been investigated in observational studies, and a recent meta-analysis showed an increase in gastric cancer risk of 150% with prolonged PPI use.^9^ Similarly, H2RA use has also been shown to increase gastric cancer risk by 40% in a recent meta-analysis.^10^ However, some of the individual studies in this meta-analysis did not adjust for important confounders, and most of them incorporated short lag times, with three not using any lag in their main analysis.^11–13^ Lag times are recommended in studies of drug–cancer associations^14^ because (a) cancer, including gastric cancer,^15^ develops over a prolonged period of time, and medications newly prescribed in the short period before cancer diagnosis are unlikely to be causative; (b) medications prescribed immediately before cancer diagnosis could reflect reverse causality, as pre-diagnostic cancer symptoms may lead to the prescription of medications.^16^ Relatively short lags are thought to be sufficient to avoid bias from reverse causation, but the relevant lag time to address the induction and latency period is unclear, and it is therefore recommended that a range of lags are used.^14^

Previous studies have raised concerns in both patients and practitioners about the use or prescribing of acid-suppressing medications.^17^ Therefore, we investigated whether PPI or H2RA use was associated with increased gastric cancer risk in two large independent population-based studies in the United Kingdom (UK). Importantly, we adjusted for a wide range of confounders, and explored the potential for reverse causation using lags of various duration.

## Methods

### Primary Care Clinical Informatics Unit database

#### Data source

The Primary Care Clinical Information Unit (PCCIU) database is a computerised primary care dataset in Scotland, capturing ~15% of the Scottish general practice (GP)-registered population.^18^ The PCCIU database collected electronic medical records between 1993 and 2011, and captured demographic information, diagnoses (using Read codes^19^), referrals, prescriptions and other information (including smoking, alcohol intake and body mass index [BMI: kg/m^2^]). Access to the data was obtained following an application to the Research Applications and Data Management Team, University of Aberdeen. Ethical approval for this study was supplied by the Queen’s University Belfast, School of Medicine and Dentistry and Biomedical Sciences Research Ethics Committee (reference number: 15.43).

#### Study design

A nested case–control study was conducted within the PCCIU database. Individuals with newly diagnosed primary gastric cancer (Read code as B11) between 1 January 1999 and 30 April 2011 were identified as cases. Up to five controls were matched to each case on age, sex and GP practice, to form a case-matched set. We defined the index date as the cancer diagnosis date in each case-matched set. Cases and controls with any previous cancer diagnoses (apart from non-melanoma skin cancer) before the index date were excluded.

In this study, the start of prescription records was from 1 January 1996 as prescriptions before this time were less likely to be recorded electronically, or the date of GP registration if this occurred after 1 January 1996. The shortest duration of prescription records was identified within each matched set. The start of the exposure period was then set as the index date minus this shortest duration within each matched set of a case and controls to ensure that all members of the matched set had an identical length of exposure period. The exposure period ended 1 year prior to the index date, to reduce the risk of reverse causality and exclude medications that are unlikely to have caused the cancer.^20^ In the main analysis, we excluded individuals who had less than 3 years of records prior to their index date. In addition, gastric cancer cases without matched controls were excluded.

#### Definition of exposure

An individual was considered a medication user based on any prescriptions in the exposure period. PPIs were esomeprazole, lansoprazole, omeprazole, pantoprazole or rabeprazole sodium; H2RAs were cimetidine, famotidine, nizatidine or ranitidine, as listed within the British National Formulary.^21^ Drug quantity and strength from prescription records were used to calculate the number of defined daily doses (DDD) for each prescription using World Health Organization methodology.^22,23^ High-dose PPI use was estimated based on the National Institute for Health and Care Excellence guidelines.^24^ We identified the high-dose PPI users if they were ever prescribed 40 mg of esomeprazole, 40 mg of rabeprazole or 40 mg of omeprazole at least once daily, or 30 mg of lansoprazole or 40 mg of pantoprazole at least twice daily.

#### Covariates

We determined lifestyle risk factors from codes in electronic GP records, using the most recent entries prior to the index date. Smoking status (never, former or current smokers), alcohol consumption (none, low, e.g. moderate or light drinker, or high intake, e.g. above recommended limits, chronic alcoholism) and obesity (BMI > 30, or not obese) were extracted. Comorbidities during the exposure period were also identified from GP records, including diabetes, coronary heart disease, myocardial infarction, heart failure, peripheral vascular disease, cerebrovascular disease, cerebrovascular accident, chronic obstructive pulmonary disease, mental illness, liver disease, oesophagitis and peptic ulcer. GP postcodes were used to estimate social deprivation on the basis of the Scottish Index of Multiple Deprivation.^25^ Any aspirin or statin use in the exposure period was identified as previous studies have shown associations between these medications and gastric cancer risk.^26–28^

#### Statistical analysis

In the PCCIU database, we used conditional logistic regression to estimate odd ratios (OR) and 95% confidence intervals (CI) for the association between PPI or H2RA use and gastric cancer risk. The matched design accounted for age, sex and GP practice; then we adjusted for obesity, aspirin and statin use and comorbidities, including diabetes, coronary heart disease, myocardial infarction, heart failure, peripheral vascular disease, cerebrovascular disease, cerebrovascular accident, chronic obstructive pulmonary disease, mental illness, liver disease, oesophagitis and peptic ulcer. Furthermore, we repeated the analysis additionally adjusting for smoking status and alcohol consumption (in individuals for whom these data were available). Dose–response analyses were conducted based on DDD and the number of prescriptions (365 DDD or 12 prescriptions approximately corresponded to 1 year of issued medication). We categorised users as 1–183 DDD, 184–365 DDD, 366–1095 DDD or more than 1095 DDD. Similarly, the number of prescriptions was divided into four categories as 1–6 prescriptions, 6–12 prescriptions, 12–36 prescriptions and more than 36 prescriptions. An analysis was conducted to investigate high-dose PPI use and gastric cancer risk as a previous study suggested that high-dose PPI may have potential anticancer properties but not low-dose PPI.^29^ We also conducted an analysis adjusting for H2RA and PPI simultaneously. An active comparator analysis was conducted by comparing PPI users with users of only H2RA in the exposure period. A similar active comparator analysis was conducted by comparing H2RA with only PPI users in the exposure period. In addition, we conducted an analysis by removing peptic ulcer and oesophagitis from the main model, as these could lie on the causal pathway. Moreover, we conducted a sensitivity analysis using multiple imputation with chained equations to adjust for missing smoking and alcohol values as the main analysis used a complete case approach.^30^ The imputation model for smoking category used ordered logit models for cases and controls separately, adjusted for age, sex, GP practice, PPI (or H2RA), obesity, comorbidities (as mentioned above), statins and aspirin. Twenty-five imputations were conducted, and the results were combined using Rubin’s rules.^31^ The same methods were utilised for alcohol imputation. We also conducted a sub-group analysis by sex using interaction terms within the models to compare associations by sex.

Sensitivity analyses were conducted by varying the duration of lag investigated as recommended.^14,32^ Specifically, we conducted a series of analyses by removing prescriptions in 2 years (including only individuals with at least 4 years of GP records), 3 years (including only individuals with at least 5 years of GP records), 4 years (including only individuals with at least 6 years of GP records) and 5 years (including only individuals with at least 7 years of GP records) prior to the index date, separately. Next, analyses were conducted by investigating medication use in 1-year intervals before gastric cancer diagnosis/index date. Specifically, we identified the users in the year before the index date (including individuals with at least 3 years of records), in the period of 1–2 years before the index date (including individuals with at least 3 years of records), in the period of 2–3 years before the index date (including individuals with at least 4 years of records), in the period of 3–4 years before the index date (including individuals with at least 5 years of records) and in the period of 4–5 years before the index date (including individuals with at least 6 years of records). In addition, we conducted an analysis comparing new use of PPI/H2RA in the year before the index date (including individuals with at least 3 years of records, to ensure at least 2 years of not using PPI/H2RA), respectively.

Finally, separate analyses, by varying the duration of lags as illustrated above, were conducted for omeprazole and lansoprazole (the most commonly used PPIs) and cimetidine and ranitidine (the most commonly used H2RAs).

### UK Biobank

#### Data source

The UK Biobank is a large prospective cohort that recruited approximately 500,000 adults aged 40–70 across England, Wales and Scotland from 2006 to 2010.^33^ At baseline, the participants completed a touchscreen questionnaire (which captured demographic data, lifestyle and environmental exposures and medical history), underwent physical measurements and provided blood and urine samples. The UK Biobank is linked to cancer registries from the Health and Social Care Information Centre (England and Wales), or the National Health Service Central Register (Scotland). The UK Biobank has ethical approval from the North West Multi-Centre Research Ethics Committee. All participants provided written informed consent.

#### Study design

During the UK Biobank cohort follow-up, newly diagnosed gastric cancer cases were identified based on the International Classification of Diseases (ICD-10) codes (C16), up to 30 September 2014. Cancer cases were further classified by histology on the basis of ICD for Oncology codes (ICD-O), as adenocarcinoma (ICD-O 8140–8573) or squamous cell carcinoma (ICD-O 8050–8082). In addition, gastric cancers were classified by anatomical site into gastric cardia cancer (C16.0) and gastric non-cardia cancer (C16.1–16.5). Individuals with previous cancer (apart from non-melanoma skin cancer) before baseline or in the year after baseline were excluded (as those cancer cases might have been present at baseline). We started the follow-up from 1 year after baseline and ended at the earliest of gastric cancer diagnosis or censoring due to death, emigration or 30 September 2014.

#### Exposure

PPI and H2RA use were self-reported at the baseline using the touchscreen questionnaire, and then verified during verbal interview with a UK Biobank nurse.

#### Covariates

Information on potential risk factors for gastric cancer was retrieved from electronic touchscreen records, collected at baseline, including smoking status (never, ever and current smoker), alcohol consumption (never, <1 day per week, 1–2 days per week, 3–4 days per week or >4 days per week), BMI (categorised as underweight or normal [<25], overweight [25–30] or obese [>30]), comorbidities (including GORD, peptic ulcer, oesophagitis and diabetes) and deprivation that was retrieved from Townsend score (based on postcode of usual residence).^34^ Other medication uses (statins and aspirin) at baseline were also ascertained.

#### Statistical analysis

In the UK Biobank, we used Cox regression models (with age as the underlying timescale) to calculate hazard ratios (HR) and 95% CI for the association between PPI/H2RA and gastric cancer risk before and after adjustment. All analyses were adjusted for age, sex, deprivation, BMI, alcohol, smoking, comorbidities at baseline (including diabetes, GORD, oesophagitis and peptic ulcer) and statins/aspirin use at baseline. Separate analyses were conducted by medication and gastric cancer subtypes (gastric adenocarcinoma, gastric cardia and gastric non-cardia).

Sensitivity analyses were conducted stratifying by sex, additionally adjusting for H2RA, additionally adjusting for the year of cohort entry, removing GORD, oesophagitis and peptic ulcer from the main model (as these could lie on the causal pathway) and by repeating the analyses using lags of 2 and 3 years by starting follow-up at 2 and 3 years after baseline, respectively.

## Results

### Primary Care Clinical Informatics Unit database

In the PCCIU database, we initially identified 1129 gastric cancer cases, after dropping 10 cases without matched controls; this study that included 1119 gastric cancer cases and 5394 matched controls, amongst 90% of the cases, was matched with five controls. The median duration of the exposure period was 5.1 (range 2.0–13.7) years for cases and controls. Generally, demographic, lifestyle and healthy characteristics were similar in gastric cancer cases and controls though a greater proportion of gastric cancer cases were former or current smokers, see Table 1 and Supplementary Table 1 for comorbidities.

**Table 1:** Characteristics of gastric cancer cases and controls in the PCCIU database and UK Biobank.

	PCCIU		UK Biobank	
	Cases, *n* (%)	Controls, *n* (%)	Gastric cancer, *n* (%)	No gastric cancer, *n* (%)
Count	1119 (17.3)	5394 (82.8)	250	471,529
Median exposure years (Min, Max)	5.1 (2.0, 13.7)	5.1 (2.0, 13.7)		
Year of diagnosis				
1996–1999	68 (6.1)	332 (6.2)		
2000–2003	330 (29.5)	1609 (29.8)		
2004–2007	494 (44.1)	2359 (43.7)	1 (0.4)	
2008–2011	227 (20.3)	1094 (20.3)	99 (39.6)	
2012–2015			150 (60.0)	
Age at index/baseline^a^				
0–59	165 (14.8)	818 (15.2)	70 (28.0)	273,444 (58.0)
60–69	292 (26.1)	1450 (26.9)	175 (70.0)	195,959 (41.6)
70+	662 (59.2)	3126 (57.9)	5 (2.0)	2126 (0.5)
Male	639 (57.1)	3082 (57.1)	182 (72.8)	217,146 (46.1)
Deprivation				
1 (least deprived)	133 (11.9)	635 (11.9)	41 (16.4)	94,422 (20.0)
2	182 (16.3)	866 (16.0)	40 (16.0)	94,009 (19.9)
3	242 (21.6)	1167 (21.6)	49 (19.6)	93,929 (19.9)
4	284 (25.4)	1378 (25.5)	55 (22.0)	94,383 (20.0)
5 (most deprived)	268 (23.9)	1300 (24.1)	64 (25.6)	94,194 (20.0)
Missing	10 (0.9)	48 (0.9)	1 (0.4)	592 (0.1)
Smoking status^b^				
Never	387 (34.6)	2052 (38.0)	99 (39.6)	257,994 (54.7)
Former	320 (28.6)	1414 (26.2)	106 (42.4)	160,790 (34.1)
Current	249 (22.2)	1009 (18.7)	41 (16.4)	50,005 (10.6)
Missing	163 (14.6)	919 (17.0)	4 (1.6)	2740 (0.6)
Selected comorbidities				
GORD			16 (6.4)	19,582 (4.2)
Peptic ulcer	15 (1.3)	75 (1.4)	7 (2.8)	5724 (1.2)
Diabetes	54 (4.8)	222 (4.1)	22 (8.8)	23,821 (5.1)
Oesophagitis	7 (0.6)	44 (0.8)	4 (1.6)	1361 (0.3)
Other drug use				
Statins	265 (23.7)	1248 (23.1)	68 (27.2)	76,473 (16.2)
Aspirin	358 (32.0)	1610 (29.9)	72 (28.8)	64,717 (13.7)
BMI				
Normal/underweight			61 (24.4)	154,912 (32.9)
Overweight			119 (47.6)	199,202 (42.2)
Obese	164 (14.7)	1060 (19.7)	70 (28.0)	114,501 (24.3)
Missing/not obese	955 (85.3)	4334 (80.4)		
Missing			0	2914 (0.6)
Alcohol consumption^2^				
Never	237 (21.2)	969 (18.0)	27 (10.8)	37,865 (8.0)
<1 day per wk			62 (24.8)	106,482 (22.6)
1–2 days per wk			58 (23.2)	121,461 (25.8)
3–4 days per wk			50 (20.0)	108,892 (23.1)
>4 days per wk			51 (20.4)	95,401 (20.2)
Low	529 (47.3)	2660 (49.3)		
High	42 (3.7)	184 (3.4)		
Missing	311 (27.8)	1581 (29.3)	2 (0.8)	1428 (0.3)

^a^Age at index date in the PCCIU database and age at baseline in the UK Biobank.

^b^Smoking status and alcohol consumption based on Read Codes in the PCCIU database and questionnaire in the UK Biobank.

Overall, a greater proportion of gastric cancer cases used PPI compared with controls (29.4% vs. 22.5%, Table 2). Use of PPI was associated with a 45% increase in the risk of gastric cancer (unadjusted OR = 1.45, 95% CI 1.25, 1.68), which was little altered after adjustment for confounders (fully adjusted OR = 1.49, 95% CI 1.24, 1.80), Table 2. The association appeared slightly more marked for females (fully adjusted OR = 1.84, 95% CI 1.38, 2.47) compared with males (fully adjusted OR = 1.26, 95% CI 0.97, 1.62); however, there was little evidence of a difference (*P* for interaction = 0.092). Dose–response analysis by DDD showed that this association was most marked for short-term use of PPI (183 or less DDD PPI vs. no use, fully adjusted OR = 1.84, 95% CI 1.43, 2.38), and was attenuated for longer-term use of PPI (1096 or more DDD vs. no use, fully adjusted OR = 1.30, 95% CI 0.91, 1.85). A similar pattern of results was found when dose–response analysis was examined by increasing the number of prescriptions. There was no evidence of an association between use of high-dose PPI and gastric cancer risk (fully adjusted OR = 1.23, 95% CI 0.76, 1.97). When lags of 2 or 3 years were used (i.e. removing prescriptions in 2 or 3 years before diagnosis), the overall association was attenuated (fully adjusted OR = 1.13, 95% CI 0.91, 1.40, and fully adjusted OR = 1.03, 95% CI 0.80, 1.33, respectively), see Table 2 and Supplementary Figures for graphical presentation. Further analysis of PPI use in specific periods before diagnosis showed that PPI was much more commonly used in gastric cancer cases compared with controls in the year before cancer diagnosis (fully adjusted OR = 7.04, 95% CI 5.57, 8.61) and in the period of 1–2 years before diagnosis (fully adjusted OR = 1.51, 95% CI 1.23, 1.84), but not before this time. Moreover, 33.6% of gastric cancer patients newly used PPI in the year before diagnosis compared with 4.4% of controls (fully adjusted OR = 10.98, 95% CI 8.47, 14.23), and this association was slightly more marked in the 55–69 age group (Supplementary Table 2).

**Table 2:** The association between PPI use and the risk of gastric cancer in the PCCIU database.

			Unadjusted	Adjusted^a^	Fully adjusted^b^
	Case, *n* (%)	Control, *n* (%)	OR (95% CI)	OR (95% CI)	OR (95% CI)
PPI main analysis (removing 1 year before index)					
PPI user vs. non-user	329/1119 (29.4)	1213/5394 (22.5)	1.45 (1.25, 1.68)	1.48 (1.26, 1.73)	1.49 (1.24, 1.80)
Male only	167/639 (26.1)	668/3082 (21.7)	1.29 (1.05, 1.58)	1.30 (1.05, 1.61)	1.26 (0.97, 1.62)
Female only	162/480 (33.8)	545/2312 (23.6)	1.66 (1.33, 2.06)	1.74 (1.38, 2.19)	1.84 (1.38, 2.47)
High-dose PPI user vs. non-user	33/1119 (3.0)	109/5394 (2.0)	1.40 (0.94, 2.10)	1.40 (0.93, 2.12)	1.23 (0.76, 1.97)
Dose–response analysis (removing 1 year before index)					
1–183 DDDs vs. non-user	141/1119 (12.6)	441/5394 (8.2)	1.74 (1.41, 2.14)	1.77 (1.43, 2.19)	1.84 (1.43, 2.38)
184–365 DDDs vs. non-user	43/1119 (3.8)	163/5394 (3.0)	1.41 (1.00, 1.99)	1.48 (1.04, 2.09)	1.49 (0.97, 2.28)
366–1095 DDDs vs. non-user	81/1119 (7.2)	352/5394 (6.5)	1.21 (0.94, 1.56)	1.23 (0.94, 1.59)	1.20 (0.89, 1.64)
≥1096 DDDs vs. non-user	64/1119 (5.7)	257/5394 (4.8)	1.32 (0.98, 1.77)	1.31 (0.97, 1.77)	1.30 (0.91, 1.85)
* P* value for the trend			0.003	0.004	0.026
1–6 prescriptions vs. non-user	153/1119 (13.7)	482/5394 (13.7)	1.71 (1.40, 2.10)	1.74 (1.42, 2.14)	1.85 (1.44, 2.37)
6–12 prescriptions vs. non-user	44/1119 (3.9)	167/5394 (3.1)	1.42 (1.01, 2.00)	1.46 (1.04, 2.07)	1.39 (0.91, 2.13)
12–36 prescriptions vs. non-user	90/1119 (8.0)	366/5394 (6.8)	1.31 (1.02, 1.68)	1.33 (1.04, 1.72)	1.35 (1.00, 1.82)
≥36 prescriptions vs. non-user	42/1119 (3.7)	198/5394 (3.7)	1.07 (0.75, 1.53)	1.06 (0.74, 1.52)	0.97 (0.64, 1.47)
* P* value for the trend			0.008	0.010	0.073
PPI user vs. non-user when removing prescriptions in specific duration					
Removing 2 years before index	212/862 (24.6)	878/4126 (21.3)	1.20 (1.00, 1.43)	1.19 (0.99, 1.43)	1.13 (0.91, 1.40)
Removing 3 years before index	137/644 (21.3)	620/3062 (20.3)	1.04 (0.84, 1.28)	1.04 (0.83, 1.29)	1.03 (0.80, 1.33)
Removing 4 years before index	87/474 (18.3)	421/2235 (18.8)	0.93 (0.71, 1.21)	0.92 (0.70, 1.21)	0.89 (0.65, 1.22)
Removing 5 years before index	55/345 (15.9)	267/1611 (16.6)	0.90 (0.65, 1.25)	0.90 (0.64, 1.26)	0.82 (0.55, 1.22)
PPI user in specific time periods before index date/cancer diagnosis date					
0–1 y before index^c^	664/1119 (59.3)	1088/5394 (20.2)	6.32 (5.44, 7.33)	6.79 (5.82, 7.91)	7.04 (5.75, 8.61)
1–2 y before index^d^	259/1119 (23.2)	926/5394 (17.2)	1.45 (1.23, 1.70)	1.47 (1.25, 1.74)	1.51 (1.23, 1.84)
2–3 y before index^e^	165/862 (19.1)	670/4126 (16.2)	1.20 (0.99, 1.46)	1.20 (0.98, 1.47)	1.15 (0.90, 1.46)
3–4 y before index^f^	107/644 (16.6)	481/3062 (15.7)	1.04 (0.82, 1.31)	1.02 (0.81, 1.30)	1.00 (0.76, 1.33)
4–5 y before index^g^	68/474 (14.4)	329/2235 (14.7)	0.93 (0.70, 1.24)	0.92 (0.69, 1.23)	0.96 (0.68, 1.35)
PPI new user vs. PPI non-new user^h^	376/1119 (33.6)	235/5394 (4.4)	10.93 (9.01,13.25)	11.11 (9.14, 13.51)	10.98 (8.47, 14.23)
Omeprazole user vs. non-user					
Removing 1 year before the index	201/1119 (18.0)	774/5394 (14.4)	1.30 (1.09, 1.55)	1.29 (1.07, 1.54)	1.21 (0.97, 1.50)
Removing 2 years before the index	123/862 (14.3)	548/4126 (13.3)	1.06 (0.85, 1.32)	1.03 (0.82, 1.29)	0.92 (0.70, 1.20)
Removing 3 years before the index	78/644 (12.1)	373/3062 (12.2)	0.95 (0.73, 1.24)	0.95 (0.72, 1.25)	0.89 (0.65, 1.23)
Lansoprazole user vs. non-user					
Removing 1 year before the index	169/1119 (15.1)	610/5394 (11.3)	1.40 (1.16, 1.69)	1.42 (1.17, 1.73)	1.49 (1.18, 1.88)
Removing 2 years before the index	114/862 (13.2)	447/4126 (10.8)	1.24 (0.99, 1.56)	1.24 (0.99, 1.57)	1.27 (0.97, 1.66)
Removing 3 years before the index	75/644 (11.7)	319/3062 (10.4)	1.11 (0.85, 1.46)	1.12 (0.84, 1.48)	1.17 (0.85, 1.62)
Sensitivity analysis (PPI user vs. non-user)					
Additionally adjusted for H2RA^i^	329/1119 (29.4)	1213/5394 (22.5)	1.45 (1.25, 1.68)	1.41 (1.20, 1.65)	1.43 (1.18, 1.73)
PPI user vs. H2RA user^j^	329/431 (76.3)	1213/1604 (75.6)	1.18 (0.85, 1.65)	1.24 (0.88, 1.75)	1.34 (0.85, 2.09)
Adjusted for lifestyle using multiple imputation^k^	329/1119 (29.4)	1213/5394 (22.5)	1.45 (1.25, 1.68)	1.48 (1.26, 1.73)	1.47 (1.26, 1.72)
Additionally not adjusted for peptic ulcer and oesophagitis^l^	329/1119 (29.4)	1213/5394 (22.5)	1.45 (1.25, 1.68)	1.45 (1.25, 1.69)	1.44 (1.20, 1.74)

^a^Study matched on age, sex and general practice, and the model contains obesity, comorbidities in the exposure period (including diabetes, coronary heart disease, myocardial infarction, heart failure, peripheral vascular disease, cerebrovascular disease, cerebrovascular accident, chronic obstructive pulmonary disease, mental illness, liver disease, peptic ulcer and oesophagitis) and other medication uses in the exposure period (statins and aspirin).

^b^Additionally adjusted for alcohol and smoking.

^c^Medication use in the year prior to diagnosis/index date restricted to individuals with at least 3 years of records.

^d^Medication use in the year from 2 to 1 year prior to diagnosis/index date restricted to individuals with at least 3 years of records.

^e^Medication use in the year from 3 to 2 years prior to diagnosis/index date restricted to individuals with at least 4 years of records.

^f^Medication use in the year from 4 to 3 years prior to diagnosis/index date restricted to individuals with at least 5 years of records.

^g^Medication use in the year from 5 to 4 years prior to diagnosis/index date restricted to individuals with at least 6 years of records.

^h^Proportion of cases and controls who used PPI in the year before diagnosis and who had not previously used PPI.

^i^Additionally adjusted for H2RA.

^j^Using only H2RA users as an active comparator.

^k^Using multiple imputation to adjust for alcohol and smoking.

^l^Removing the peptic ulcer and oesophagitis adjustment from the main model.

Similarly, H2RA use was associated with an increase in the risk of gastric cancer (fully adjusted OR = 1.44, 95% CI 1.16, 1.80, see Table 3). Similar associations were observed in stratified analysis by sex (fully adjusted OR = 1.43, 95% CI 1.05, 1.94 in men, and fully adjusted OR = 1.45, 95% CI 1.04, 2.01 in women, respectively). No clear dose response was observed as increases in risk were seen even for short-term use (183 or less DDD H2RA vs. never use, fully adjusted OR = 1.40, 95% CI 1.05, 1.85). The H2RA association was slightly attenuated when removing prescriptions in 2 years before diagnosis, and largely attenuated when 3 years were removed (fully adjusted OR = 1.32, 95% CI 1.02, 1.70, and fully adjusted OR = 1.16, 95% CI 0.85, 1.57, respectively). H2RA was more commonly used and associated with an increased risk of gastric cancer in the period of 1 year before diagnosis (fully adjusted OR = 3.07, 95% CI 2.37, 3.98), 1–2 years before diagnosis (fully adjusted OR = 1.87, 95% CI 1.40, 2.50) and 2–3 years before diagnosis (fully adjusted OR = 1.55, 95% CI 1.11, 2.16), but not before this time. Overall, 7.9% of gastric cancer patients newly used H2RA in the year before diagnosis compared with 1.1% of controls (fully adjusted OR = 9.87, 95% CI 6.04, 16.15), and this association was more marked in the group aged younger than 55 years (Supplementary Table 2).

**Table 3:** The association between H2RA use and the risk of gastric cancer in the PCCIU database.

			Unadjusted	Adjusted^a^	Fully adjusted^b^
	Case, *n* (%)	Control, *n* (%)	OR (95% CI)	OR (95% CI)	OR (95% CI)
H2RA main analysis (removing 1 year before the index)					
H2RA user vs. non-user	199/1119 (17.8)	689/5394 (12.8)	1.49 (1.25, 1.77)	1.49 (1.24, 1.78)	1.44 (1.16, 1.80)
Male only	100/639 (15.6)	356/3082 (11.6)	1.42 (1.11, 1.82)	1.41 (1.10, 1.81)	1.43 (1.05, 1.94)
Female only	99/480 (20.6)	333/2312 (14.4)	1.56 (1.21, 2.01)	1.57 (1.21, 2.04)	1.45 (1.04, 2.01)
Dose–response analysis (removing 1 year before the index)					
1–183 DDDs vs. non-user	107/1119 (9.6)	370/5394 (6.9)	1.49 (1.18, 1.87)	1.49 (1.18, 1.88)	1.40 (1.05, 1.85)
184–365 DDDs vs. non-user	22/1119 (2.0)	76/5394 (1.4)	1.50 (0.93, 2.41)	1.49 (0.92, 2.40)	1.42 (0.77, 2.60)
366–1095 DDDs vs. non-user	51/1119 (4.6)	172/5394 (3.2)	1.52 (1.10, 2.10)	1.51 (1.09, 2.10)	1.50 (0.99, 2.29)
≥1096 DDDs vs. non-user	19/1119 (1.7)	71/5394 (1.3)	1.38 (0.82, 2.33)	1.37 (0.81, 2.32)	1.62 (0.86, 3.05)
* P* value for the trend			<0.001	<0.001	0.003
1–6 prescriptions vs. non-user	105/1119 (9.4)	375/5394 (6.9)	1.44 (1.14, 1.81)	1.44 (1.14, 1.82)	1.38 (1.04, 1.83)
6–12 prescriptions vs. non-user	34/1119 (3.0)	101/5394 (1.8)	1.72 (1.16, 2.56)	1.71 (1.15, 2.55)	1.54 (0.93, 2.54)
12–36 prescriptions vs. non-user	46/1119 (4.1)	163/5394 (3.0)	1.46 (1.04, 2.05)	1.45 (1.03, 2.05)	1.58 (1.03, 2.42)
≥36 prescriptions vs. non-user	14/1119 (1.3)	50/5394 (0.9)	1.44 (0.79, 2.63)	1.44 (0.79, 2.64)	1.34 (0.62, 2.90)
* P* value for the trend			<0.001	<0.001	0.003
H2RA user vs. non-user when removing prescriptions in specific duration					
Removing 2 years before the index	137/862 (15.9)	518/4126 (12.6)	1.32 (1.07, 1.63)	1.30 (1.05, 1.61)	1.32 (1.02, 1.70)
Removing 3 years before the index	93/644 (14.4)	386/3062 (12.6)	1.16 (0.90, 1.50)	1.15 (0.89, 1.48)	1.16 (0.85, 1.57)
Removing 4 years before the index	63/474 (13.3)	284/2235 (12.7)	1.03 (0.76, 1.39)	1.01 (0.74, 1.37)	1.06 (0.74, 1.52)
Removing 5 years before the index	44/345 (12.8)	199/1611 (12.4)	1.00 (0.70, 1.43)	0.99 (0.69, 1.43)	1.13 (0.74, 1.72)
H2RA user in specific time periods before index date/cancer diagnosis date					
0–1 y before index^c^	178/1119 (15.9)	323/5394 (6.0)	3.05 (2.49, 3.72)	3.04 (2.48, 3.72)	3.07 (2.37, 3.98)
1–2 y before index^d^	114/1119 (10.2)	329/5394 (6.1)	1.77 (1.42, 2.22)	1.78 (1.41, 2.23)	1.87 (1.40, 2.50)
2–3 y before index^e^	77/862 (8.9)	263/4126 (6.4)	1.46 (1.12, 1.91)	1.45 (1.11, 1.91)	1.55 (1.11, 2.16)
3–4 y before index^f^	53/644 (8.4)	196/3062 (6.4)	1.32 (0.96, 1.83)	1.33 (0.96, 1.85)	1.26 (0.85, 1.87)
4–5 y before index^g^	38/474 (8.0)	136/2235 (6.1)	1.35 (0.92, 1.98)	1.34 (0.91, 1.97)	1.29 (0.81, 2.05)
H2RA new user vs. H2RA non-new user^h^	89/1119 (7.9)	58/5394 (1.1)	8.11 (5.75, 11.43)	8.26 (5.85, 11.68)	9.87 (6.04, 16.15)
Cimetidine user vs. non-user					
Removing 1 year before the index	85/1119 (7.6)	254/5394 (4.7)	1.68 (1.30, 2.17)	1.66 (1.28, 2.15)	1.43 (1.02, 2.01)
Removing 2 years before the index	62/862 (7.2)	193/4126 (4.7)	1.59 (1.18, 2.15)	1.55 (1.14, 2.10)	1.31 (0.90, 1.93)
Removing 3 years before the index	39/644 (6.1)	148/3062 (4.8)	1.27 (0.88, 1.83)	1.23 (0.85, 1.79)	1.10 (0.70, 1.75)
Ranitidine user vs. non-user					
Removing 1 year before the index	128/1119 (11.4)	459/5394 (8.5)	1.39 (1.12, 1.71)	1.10 (0.94, 1.29)	1.42 (1.10, 1.85)
Removing 2 years before the index	78/862 (9.1)	341/4126 (8.3)	1.09 (0.84, 1.42)	1.09 (0.84, 1.43)	1.22 (0.89, 1.67)
Removing 3 years before the index	57/644 (8.9)	255/3062 (8.3)	1.05 (0.77, 1.43)	1.05 (0.77, 1.44)	1.17 (0.81, 1.68)
Sensitivity analysis (H2RA user vs. non-user)					
Additionally adjusted for PPI^i^	199/1119 (17.8)	689/5394 (12.8)	1.49 (1.25, 1.77)	1.39 (1.16, 1.67)	1.33 (1.07, 1.67)
H2RA user vs. PPI user^j^	199/431 (46.2)	689/1604 (43.0)	1.03 (0.78, 1.36)	1.00 (0.75, 1.33)	0.86 (0.60, 1.24)
Adjusted for lifestyle using multiple imputation^k^	199/1119 (17.8)	689/5394 (12.8)	1.49 (1.25, 1.77)	1.49 (1.24, 1.78)	1.45 (1.22, 1.75)
Additionally not adjusted for peptic ulcer and oesophagitis^l^	199/1119 (17.8)	689/5394 (12.8)	1.49 (1.25, 1.77)	1.47 (1.23, 1.76)	1.42 (1.14, 1.77)

^a^Study matched on age, sex and general practice, and the model contains obesity, comorbidities in the exposure period (including diabetes, coronary heart disease, myocardial infarction, heart failure, peripheral vascular disease, cerebrovascular disease, cerebrovascular accident, chronic obstructive pulmonary disease, mental illness, liver disease, peptic ulcer and oesophagitis) and other medication uses in the exposure period (statins and aspirin).

^b^Additionally adjusted for alcohol and smoking.

^c^Medication use in the year prior to diagnosis/index date restricted to individuals with at least 3 years of records.

^d^Medication use in the year from 2 to 1 year prior to diagnosis/index date restricted to individuals with at least 3 years of records.

^e^Medication use in the year from 3 to 2 years prior to diagnosis/index date restricted to individuals with at least 4 years of records.

^f^Medication use in the year from 4 to 3 years prior to diagnosis/index date restricted to individuals with at least 5 years of records.

^g^Medication use in the year from 5 to 4 years prior to diagnosis/index date restricted to individuals with at least 6 years of records.

^h^Proportion of cases and controls who used H2RA in the year before diagnosis, and who had not previously used H2RA.

^i^Additionally adjusted for PPI.

^j^Using only PPI users as an active comparator.

^k^Using multiple imputation to adjust for alcohol and smoking.

^l^Removing the peptic ulcer and oesophagitis adjustment from the main model.

PPI and H2RA associations were generally similar by medication subtype, after additional adjustment for each other, when not adjusting for peptic ulcer disease and oesophagitis, and when adjusting for lifestyle factors using multiple imputation, Tables 2 and 3. Null associations were observed when using an active comparator analysis (fully adjusted OR = 1.34, 95% CI 0.85, 2.09 in PPI users when using only H2RA users as an active comparator, and fully adjusted OR = 0.86, 95% CI 0.60, 1.24 in H2RA users when using only PPI users as an active comparator, respectively), see Tables 2 and 3.

### UK Biobank

There were 502,543 participants in the UK Biobank cohort, 26,869 were removed because they developed cancer before the first year after baseline and 3898 were removed because they died within the first year, leaving 471,779 in the final cohort for analysis, of whom 250 were diagnosed with gastric cancer during the median follow-up of 4.6 years (interquartile range: 3.9–5.3 years). Those who were diagnosed with gastric cancer were more likely to be older, male, from deprived areas, smoke, be overweight or obese, have comorbidities and use statins and aspirin (Table 1).

There was an increase in gastric cancer risk with PPI use (unadjusted HR = 1.53, 95% CI 1.10, 2.12), but this risk was attenuated after adjustment for confounders (adjusted HR = 1.28, 95% CI 0.86, 1.90, see Table 4). These associations were attenuated when using a lag of 2 years by starting follow-up at 2 years after baseline (unadjusted HR = 1.28, 95% CI 0.86, 1.89, and adjusted HR = 1.15, 95% CI 0.73, 1.82).

**Table 4:** The association between PPI or H2RA use and the risk of gastric cancer in the UK Biobank.

	Users		Non-users		Unadjusted HR (95% CI)	Adjusted^a^ HR (95% CI)
	Gastric cancer, *n*	Person-years	Gastric cancer, *n*	Person-years		
PPI user vs. non-user						
Main analysis (starting follow-up at 1 y)	44	208,807	206	1,949,341	1.53 (1.10, 2.12)	1.28 (0.86, 1.90)
Male only	29	94,195	153	896,467	1.43 (0.96, 2.13)	1.14 (0.70, 1.87)
Female only	15	114,611	53	1,052,874	1.94 (1.09, 3.47)	1.73 (0.86, 3.45)
Adenocarcinoma	37	208,807	175	1,949,341	1.52 (1.07, 2.18)	1.18 (0.76, 1.83)
Gastric cardia	15	208,807	86	1,949,341	1.26 (0.72, 2.18)	0.81 (0.40, 1.64)
Gastric non-cardia	14	208,807	51	1,949,341	1.93 (1.06, 3.50)	1.44 (0.68, 3.06)
Main additionally adjusting for H2RA^b^	44	208,807	206	1,949,341	1.53 (1.10, 2.12)	1.26 (0.84, 1.88)
Main removing adjustment for GORD, oesophagitis and peptic ulcer^c^	44	208,807	206	1,949,341	1.53 (1.10, 2.12)	1.41 (1.00, 1.98)
Main additionally adjusting for year of cohort entry^d^	44	208,807	206	1,949,341	1.53 (1.10, 2.12)	1.28 (0.86, 1.90)
Starting follow-up at 2 y	30	162,955	170	1,525,464	1.28 (0.86, 1.89)	1.15 (0.73, 1.82)
Starting follow-up at 3 y	22	117,731	122	1,105,366	1.28 (0.81, 2.02)	1.12 (0.65, 1.92)
Omeprazole user vs. non-user						
Main analysis (starting follow-up at 1 y)	25	122,860	225	2,035,288	1.43 (0.95, 2.17)	1.17 (0.74, 1.85)
Lansoprazole user vs. non-user						
Main analysis (starting follow-up at 1 y)	16	73,848	234	2,084,299	1.49 (0.90, 2.48)	1.21 (0.71, 2.08)
H2RA user vs. non-user						
Main analysis (starting follow-up at 1 y)	4	38,517	246	2,119,632	0.80 (0.30, 2.15)	0.49 (0.16, 1.56)

^a^Adjusted for age at baseline, sex, socioeconomic status, alcohol, smoking, BMI, comorbidities at baseline (including diabetes, GORD, oesophagitis and peptic ulcer) and other medication uses at baseline (statins aand aspirin).

^b^Additionally adjusted for H2RA.

^c^Removing the GORD, oesophagitis and peptic ulcer adjustment from the main model.

^d^Additionally adjusted for year of cohort entry.

The association for PPI use appeared more marked for non-cardia gastric cancer compared with cardia gastric cancer before adjustment (unadjusted HR = 1.93, 95% CI 1.06, 3.50, and HR = 1.26, 95% CI 0.72, 2.18, respectively) but not after adjustment (adjusted HR = 1.44, 95% CI 0.68, 3.06, and HR = 0.81, 95% CI 0.40, 1.64, respectively). The association was similar for gastric adenocarcinoma, by medication subtype, after additional adjustment for H2RA and after additional adjustment for year of cohort entry, but slightly less marked for males, whilst more marked when not adjusting for GORD, oesophagitis and peptic ulcer, see Table 4.

There was no evidence of an increase in gastric cancer risk with H2RA use (adjusted HR = 0.49, 95% CI 0.16, 1.56), but the numbers of H2RA use were small precluding further analysis.

## Discussion

In both the PCCIU case–control and UK Biobank cohort studies, we observed little consistent evidence of an increased risk of gastric cancer with PPI use. Although using a 1-year lag there was an association between PPI and gastric cancer, this association did not follow an exposure response (for instance, those using for the shortest period had the highest risk) and was attenuated with longer lags suggesting the role of reverse causation (for instance, associations weakened when prescriptions in the 2-year period prior to diagnosis were removed in the PCCIU, and incident gastric cancers within 2 years after baseline were removed in the UK Biobank). A similar pattern of association was observed in PCCIU for H2RA, but there was no association between H2RA use and gastric cancer in the UK Biobank.

Our PPI findings contrast with the most recent meta-analysis, in which a marked increase in gastric cancer risk with prolonged PPI use was observed with a pooled OR of 2.5 in seven observational studies.^9^ However, there was marked heterogeneity in the observed associations with odds ratios varying from 1.5 to 24.1 across the seven studies, and an earlier meta-analysis observed a less marked association for any PPI use (pooled OR of 1.4).^10^ Our findings are more consistent with this earlier meta-analysis. In our study, the association between PPI use and gastric cancer risk was sensitive to the lag-time duration, but the optimal biologically relevant lag time is unclear. In our main analysis, we used a lag time of 1 year, which assumes that PPI would take at least 1 year to induce a gastric cancer and for it to develop to the point of detection. Should this process take longer, then extended lag times would be more appropriate. Previous studies have also observed that the association between PPI use and gastric cancer risk is reduced with longer lag times. For instance, a Canadian study observed a marked association between PPI and gastric cancer (OR of 2.9), but this was attenuated with a 2-year lag time (OR of 1.2).^35^ Also, a Swedish cohort study observed a marked association (standardised incidence ratio [SIR] of 3.4) that was attenuated after excluding cancers in the year after medication started (SIR = 1.6).^11^ Similarly, a UK study observed an association between PPI and gastric cancer risk only in current users who used in the year before diagnosis.^36^ Any difference in our findings and the previous meta-analyses could reflect ethnic differences, as two studies were based on Asian populations and investigated PPI in the context of *H. pylori* eradication,^13,37^ or confounding as some studies had limited confounders such as alcohol.^11^

Of relevance, two systematic reviews of RCTs of PPI found no evidence that PPI could cause or accelerate the development of the premalignant gastric lesions, atrophic gastritis and intestinal metaplasia, but the numbers of included events were small.^38,39^

Our H2RA findings are similar to a meta-analysis that observed a pooled OR of 1.4 in ten observational studies.^10^ The less marked association in our study could reflect better adjustment for confounders in our study, or the inclusion of low-quality observational studies in that review, both of which were shown to influence the pooled OR with more recent studies and studies of higher quality observing less marked estimates.

In our study, around one-third of gastric cancer patients newly used PPI in the year before cancer diagnosis, similar but lower than that seen in the latest Swedish study.^40^ This should raise the question in the mind of the clinician prescribing a first course of PPI: could these dyspepsia symptoms be a signal of early gastric cancer?^41^ Recommending further action on the basis of new use of PPI alone however does not appear to be warranted because PPI is very widely prescribed, and this approach would only capture a third of gastric cancer patients. However, since early detection is a key determinant of survival in gastric cancer, it is possible that future research could investigate new use of acid-suppression therapy along with other factors to identify those at the highest risk.

The main strength of our study is to utilise high-quality population-based data from two independent sources. In PCCIU, medication use was determined from GP prescription records avoiding recall bias and providing detailed information on the timing, dose and quantities prescribed. In the UK Biobank, cancer outcome was determined from cancer registry records, providing verified information on tumour histology and location. In addition, in both analyses, we were able to adjust for a wide range of covariates, including many of the main risk factors for gastric cancer, such as age, sex, deprivation, smoking, BMI, alcohol consumption, relevant comorbidities (including peptic ulcer, oesophagitis in PCCIU and GORD, peptic ulcer and oesophagitis in the UK Biobank) and medication use (including statins and aspirin).

Several limitations of our study must be acknowledged. First, in the UK Biobank, medication use was based on self-report, and even though this was verified by nurses, we could not obtain the dose or the frequency of medication use. Second, the UK Biobank has been shown to be healthier than the general population;^42^ however, aetiological findings from the UK Biobank appear to be generalisable to the UK population.^43^ Although some inaccuracy in identifying gastric cancer in GP records from PCCIU is inevitable, in general, the recording of cancer outcomes within UK GP records has been shown to be fairly accurate.^44^ Another potential limitation was confounding by indication as despite having a wide range of comorbidities, we were not able to adjust for *H. pylori*, an important risk factor for gastric cancer.^45^

To conclude, we found some evidence of the associations between PPI and H2RA use and gastric cancer risk in a large population-based case–control and a cohort study. These associations were sensitive to the duration of lag time used in the analysis. Our results revealed a marked increase in the prescription of acid-suppression medications immediately before gastric cancer diagnosis suggesting the role of reverse causation.

## Supplementary information

Supplementary information

## Data Availability

The UK Biobank data (https://www.ukbiobank.ac.uk/) and PCCIU data (https://www.abdn.ac.uk/iahs/research/primary-care/pcciur/) are available, following the access procedures, for researchers to access to conduct health-related research in the public interest.
