# Supplementary material for: Use of proton pump inhibitors and histamine-2 receptor antagonists and risk of gastric cancer in two population-based studies
Source: Br J Cancer. 2020 May 5;123(2):307–15. doi: 10.1038/s41416-020-0860-4 (PMC7374738; doi:10.1038/s41416-020-0860-4)

**Supplementary information:**

This document contains supplementary figures and legends as well as supplementary tables.

**Supplementary Tables:**

| Supplementary Table 1. Comorbidities in gastric cancer cases and controls in PCCIU database and UK Biobank. | | | | | |
| --- | --- | --- | --- | --- | --- |
|  | **PCCIU** | |  | **UK biobank** | |
|  | **Cases, n(%)** | **Controls, n (%)** |  | **Gastric cancer,**  **n (%)** | **No gastric cancer,**  **n (%)** |
| **Count** | 1119 (17.3) | 5394 (82.7) |  | 250 | 471529 |
| **Selected comorbidities** |  |  |  |  |  |
| GORD^1^ |  |  |  | 16 (6.4) | 19582 (4.2) |
| Peptic ulcer | 15 (1.3) | 75 (1.4) |  | 7 (2.8) | 5724 (1.2) |
| Diabetes | 54 (4.8) | 222 (4.1) |  | 22 (8.8) | 23821 (5.1) |
| Oesophagitis | 7 (0.6) | 44 (0.8) |  | 4 (1.6) | 1361 (0.3) |
| Coronary heart disease | 58 (5.2) | 263 (4.9) |  |  |  |
| Myocardial infarction | 24 (2.1) | 98 (1.8) |  |  |  |
| Heart failure | 27 (2.4) | 107 (2.0) |  |  |  |
| Peripheral vascular disease | 24 (2.1) | 97 (1.8) |  |  |  |
| Mental illness | 68 (6.1) | 322 (6.0) |  |  |  |
| Cerebrovascular disease | 46 (4.1) | 203 (3.7) |  |  |  |
| Cerebrovascular accident | 22 (2.0) | 88 (1.6) |  |  |  |
| Chronic obstructive pulmonary disease | 51 (4.6) | 176 (3.2) |  |  |  |
| Liver disease | 3 (0.3) | 10 (0.2) |  |  |  |
| ^1^ GORD: gastro-oesophageal reflux disease. | | | | | |

| Supplementary Table 2. The association between drug new use in the year before index date and the risk of gastric cancer in PCCIU database. | | | | | |
| --- | --- | --- | --- | --- | --- |
|  |  |  | Unadjusted | Adjusted^1^ | Fully adjusted^2^ |
|  | Case, n(%) | Control, n(%) | OR (95%CI) | OR (95%CI) | OR (95%CI) |
| PPI new user vs. non-user | 376/1119 (33.4) | 235/5394 (4.4) | 10.93 (9.01, 13.25) | 11.11 (9.14, 13.51) | 10.98 (8.47, 14.23) |
| Age at index |  |  |  |  |  |
| <55 | 40/97 (41.2) | 20/493 (4.06) | 15.77 (8.05, 30.92) | 17.11 (8.32, 35.18) | 14.72 (5.70, 37.99) |
| 55-69 | 146/360 (40.6) | 63/1775 (3.6) | 19.58 (13.32, 28.80) | 21.10 (14.18, 31.38) | 18.14 (11.13, 29.57) |
| 70+ | 190/662 (28.7) | 152/3126 (4.9) | 7.56 (5.92, 9.65) | 7.84 (6.10, 10.07) | 8.03 (5.71, 11.31) |
|  |  |  |  |  |  |
| H2RA new user vs. non-user | 89/1119 (7.9) | 58/5394 (1.1) | 8.11 (5.75, 11.43) | 8.26 (5.85, 11.68) | 9.87 (6.04, 16.15) |
| Age at index |  |  |  |  |  |
| <55 | 14/97 (14.4) | 5/493 (1.0) | 20.32 (5.80, 71.23) | 24.22 (6.24, 94.06) | 48.34 (4.59, 509.16) |
| 55-69 | 38/360 (10.6) | 24/1775 (1.4) | 8.52 (4.94, 14.71) | 8.81 (5.00, 15.15) | 9.40 (4.38, 20.14) |
| 70+ | 37/662 (5.6) | 29/3126 (1.0) | 6.07 (3.72, 9.89) | 6.33 (3.87, 10.35) | 7.88 (3.89, 15.95) |
| ^1^ Study matched on age, sex and general practice and model contains obesity, comorbidities in exposure period (including diabetes, coronary heart disease, myocardial infarction, heart failure, peripheral vascular disease, cerebrovascular disease, cerebrovascular accident chronic obstructive pulmonary disease, mental illness, liver disease, peptic ulcer, oesophagitis) and other medication use in exposure period (statins, aspirin). ^2^ Additionally adjusted for alcohol and smoking. | | | | | |

**Supplementary Figures and legends:** Graphical presentation of analyses which were conducted varying the duration of lag.

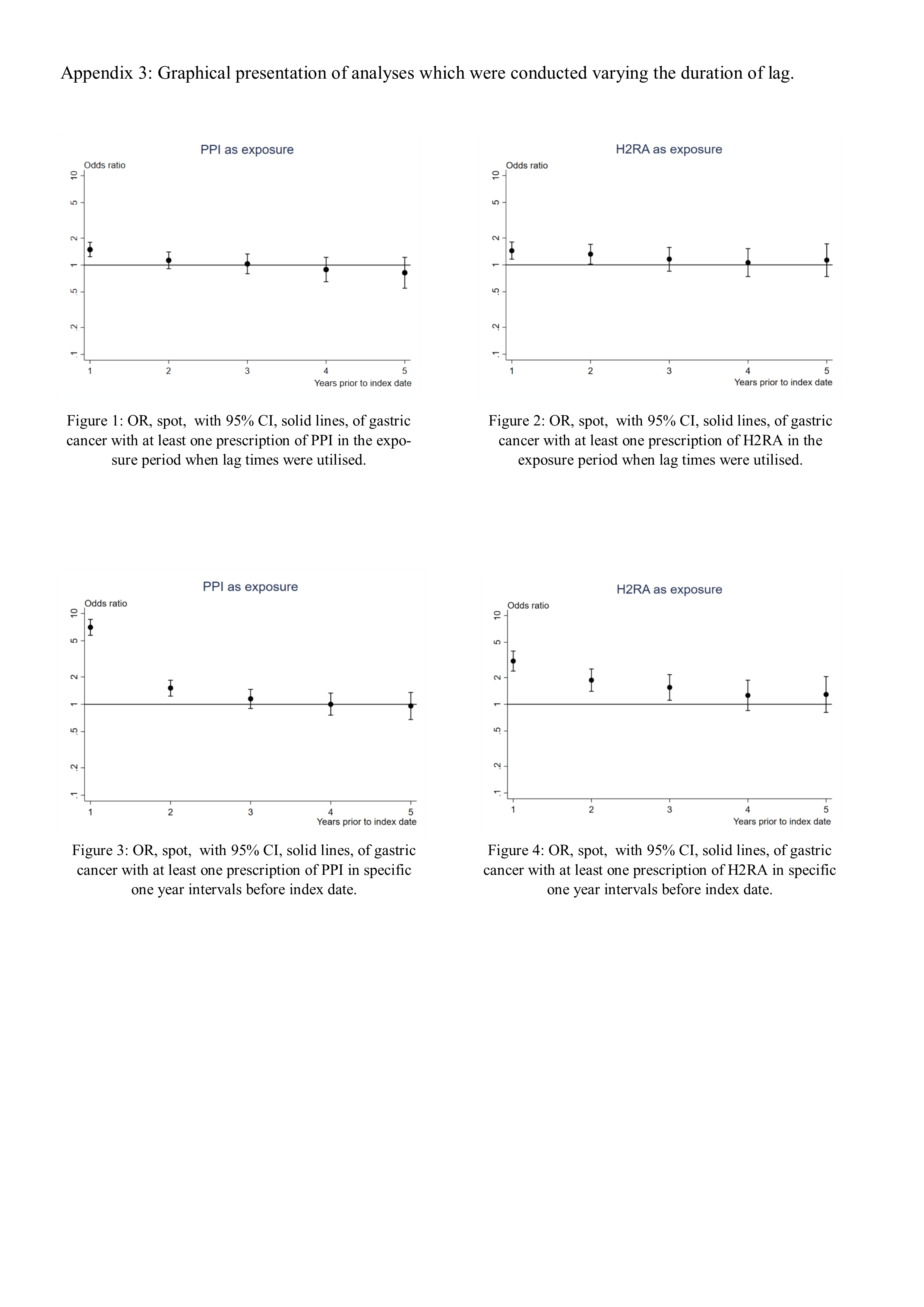

Supplement: Supplementary file 1 — Supplementary information [file 41416_2020_860_MOESM1_ESM.docx]
